# C-Peptide-Positive, Autoantibody-Negative Type 1 Diabetes Mellitus

**DOI:** 10.7759/cureus.28360

**Published:** 2022-08-24

**Authors:** Rachel M Fenner, Samantha Bookbinder, Ravi Kant, Brewer Eberly

**Affiliations:** 1 Endocrinology and Diabetes, Medical University of South Carolina, Charleston, USA; 2 Endocrinology, Medical University of South Carolina, Charleston, USA; 3 Endocrinology, Diabetes, and Metabolism, Medical University of South Carolina, Anderson, USA; 4 Family Medicine, AnMed, Anderson, USA

**Keywords:** type 1 diabetes mellitus (t1dm), type 1 diabetes mellitus immunology, pancreatic beta cells, c-peptide level, endocrinology and diabetes

## Abstract

A 30-year-old female previously diagnosed with C-peptide (CP)-positive, autoantibody-negative type 1 diabetes mellitus (T1DM) at 19 years old presented to the clinic at age 28 for management of diabetes mellitus (DM) that had previously been controlled by insulin since diagnosis. Laboratory results from May 2011 showed low-normal C-peptide of 1 ng/mL (normal range: 0.8-4 ng/mL) with no corresponding glucose, glutamic acid decarboxylase (GAD)-65 antibody (GADA) of <1 U/mL (N<1.1 U/mL at the time of laboratory draw), and HbA1c of 6.4%. Almost 10 years later, in December 2020, laboratory results showed normal C-peptide of 2.1 ng/mL with a glucose of 198 mg/dL, GAD-65 antibody of 38.2 U/mL (current reference range: 0-5 U/mL), negative pancreatic islet antibody (IA), and undetectable zinc transporter 8 (ZnT8) antibody, consistent with a diagnosis of T1DM.

This increase in CP indicates the possibility of pancreatic beta cell regeneration and/or increased function. The commonly accepted belief that individuals with T1DM quickly lose all function of pancreatic beta cells has led to academic consequences; many immunotherapy clinical trials’ inclusion criteria require participants to have a new diagnosis of T1DM based on the assumption that those with a longer duration of diabetes have unrecoverable cessation of insulin secretion. CP could influence inflammation, microvascular circulation, and endothelial function. Further, it could affect the neuronal and glomerular structure and/or function. These potential functions of CP are seen by the correlation between measurable CP levels and decreased diabetic complication rates.

## Introduction

Type 1 diabetes mellitus (T1DM) is a disease with a prevalence of nearly one in 300 in the United States by the age of 18 according to epidemiology reports, with an increasing incidence rate worldwide [1]. Patients with T1DM classically have autoimmune-mediated destruction of pancreatic beta cells, leading to insulin deficiency. It has been estimated that over 90% of newly diagnosed T1DM will have a measurable amount of antibodies, with the most common autoantibodies being glutamic acid decarboxylase (GAD)-65 antibody (GADA), islet antibody (IA)-2 antibody (IA-2A), zinc transporter 8 (ZnT8) antibody, or insulin autoantibody (IAA) [2].

Since C-peptide (CP) is co-secreted with insulin by the pancreas, C-peptide measurement is used to quantify endogenous insulin production. Until recently, it was believed that insulin and C-peptide production stopped within years of the diagnosis of T1DM [3]. However, new research has shown that C-peptide production can persist for decades following T1DM diagnosis [4]. Additionally, patients with residual C-peptide may have fewer complications.

This case study discusses a patient with increased C-peptide laboratory levels nearly a decade after the initial diagnosis of T1DM and limited morbidity. This study then discusses the implications of these findings.

## Case presentation

A 30-year-old female was initially diagnosed at the age of 19 in 2011 with CP-positive, autoantibody-negative diabetes mellitus (DM) when she presented with a month-long duration of 30-lb weight loss, an HbA1c of 13.7%, and a negative family history of DM. Laboratory results from May 2011 showed low-normal C-peptide of 1 ng/mL (normal range: 1.1-4.4 ng/mL) with no corresponding glucose, GAD-65 antibody of <1 U/mL (N<1.1 U/mL at the time of laboratory draw), and an HbA1c of 6.4%.

The patient was treated with multiple daily injections of insulin for nine years prior to establishing care at our clinic in 2020 at the age of 28. Her diabetes had been uncomplicated, but the patient was concerned about labile blood glucose levels.

During the patient’s initial presentation to our clinic in December 2020, her medications included Lantus (insulin glargine) 15 units at bedtime and Humalog (insulin lispro) 1-10 units sliding scale three times daily. She reported previous use of insulin pumps, which included Medtronic and Omnipod, and also the use of a personal continuous glucose monitor (CGM) Dexcom. She stated that she was interested in restarting an insulin pump due to a change in her financial status. At that time, the patient reported variable fasting blood sugars, including values in the low 40s that woke her up at night, as well as values in the 200s. Her average blood glucose level was 142 mg/dL with values below 54 mg/dL for 0% of the time, values 54-69 mg/dL for 1% of the time, values 70-180 mg/dL for 78% of the time, values 181-250 mg/dL for 17% of the time, and values over 250 mg/dL for 4% of the time. The patient denied polyuria, polydipsia, abdominal pain, nausea, vomiting, numbness and tingling of feet, recent vision changes, or leg cramps on exertion. Following this visit, laboratory results showed normal C-peptide of 2.1 ng/mL (normal range: 1.1-4.4 ng/mL) with a glucose of 198 mg/dL, positive GAD-65 antibody of 38.2 U/mL (current reference range: 0-5 U/mL), negative pancreatic islet antibody (ICA), and negative ZnT8 antibody, consistent with the diagnosis of T1DM.

Subsequently, the patient was switched from multiple daily injections (MDI) of insulin therapy to continuous subcutaneous insulin infusion (CSII) with Dexcom G6 and using Humalog as the insulin. In April 2021, her glycated hemoglobin (A1c) was at goal, 6.6% with minimal hypoglycemia noted per CGM. Her average blood glucose level was 148 mg/dL with values below 54 mg/dL <1% of the time, values 54-69 mg/dL <2% of the time, values 70-180 mg/dL 77% of the time, values 181-250 mg/dL 20% of the time, and values over 250 mg/dL 1% of the time. The patient reported no changes in symptoms. Table 1 shows the percentage of time in variable blood glucose ranges.

**Table 1:** Percentage of time in variable blood glucose ranges

	December 2020	April 2021	August 2021
<54 mg/dL	0%	<1%	<1%
54-69 mg/dL	1%	<2%	<2%
70-180 mg/dL	78%	77%	83%
181-250 mg/dL	17%	20%	17%
>250 mg/dL	4%	1%	<1%
Average blood sugar (mg/dL)	142	148	145

## Discussion

At the time of diagnosis in 2011, the patient had a low-normal CP level, and nearly a decade later, in 2020, CP levels increased to a midrange normal level. This increase in CP indicates the possibility of pancreatic beta cell regeneration and/or increased function. A study completed on mice showed that alpha cells could differentiate into beta cells over extended periods of time in the setting of diabetes [5]. Another study similarly found this differentiation, specifically without proliferation of cells, in the setting of mice with removed beta cells without autoantibodies [6]. This idea of the potential for an increase in number or function is further supported in human studies, such as a study on 90 pregnant women with T1DM, which found an increase in CP that reverted to baseline levels after delivery [7].

The commonly accepted belief that individuals with T1DM quickly lose all function of pancreatic beta cells has led to clinical consequences. Many immunotherapy-based clinical trials have inclusion criteria that require participants to have a new diagnosis of T1DM based on the assumption that those with a longer duration of diabetes have unrecoverable cessation of insulin secretion. This leads to a large population being excluded from research that could possibly benefit greatly. A study found that individuals with T1DM with any measurable levels of CP had a linear relationship between glycemic and CP levels and therefore functional pancreatic beta cells [8]. This leads to the question of whether CP levels in T1DM constantly fluctuate throughout time depending on coinciding factors such as blood glucose levels if functional beta cells are present.

Our patient had limited long-term consequences from her 10-year history of T1DM, which could possibly be associated with her midrange normal levels of CP, 10 years following diagnosis. There has been speculation that CP could have an influence on inflammation, microvascular circulation, and endothelial function. Further, it could affect the neuronal and glomerular structure and/or function. These potential functions of CP are seen by the correlation between measurable CP levels and decreased diabetic complication rates. A study conducted on 1,272 patients with T1DM found a statistically significant relationship between CP and complications [9]. Specifically, CP levels greater than 10 pmol/L were associated with lower rates of nephropathy, neuropathy, foot ulcers, and retinopathy. They also found that low CP was associated with poorly managed T1DM as indicated by HbA1c. Additionally, they found a relationship between low CP and episodes of hypoglycemia.

Likewise, scientists could reexamine the current dichotomous classification of type 1 and type 2 diabetes as entirely separate entities. This patient showed antibodies that target pancreatic destruction fitting the criteria for T1DM. However, the patient still produces normal amounts of C-peptide, implying that the insulin produced is insufficient at controlling blood glucose levels, such as T2DM.

## Conclusions

This case is an example of a patient with increased CP production nearly a decade after the diagnosis of T1DM. Further studies should explore the possibility of pancreatic beta cell regeneration and improvement in functionality, and immunotherapy clinical trials targeting those with retained beta cells despite prolonged T1DM could benefit a large population of individuals who currently have limited treatment options. Higher CP levels are associated with lower rates of complications such as nephropathy, neuropathy, foot ulcers, and retinopathy. Hence, exploration of the mechanism of these improved outcomes could be used to find ways to lower morbidity associated with DM.
